# Pain during Removal of Carious Lesions in Children: A Randomized Controlled Clinical Trial

**DOI:** 10.1155/2013/896381

**Published:** 2013-12-04

**Authors:** Lara Jansiski Motta, Sandra Kalil Bussadori, Ana Paula Campanelli, André Luis da Silva, Thays Almeida Alfaya, Camila Haddad Leal de Godoy, Maria Fidela de Lima Navarro

**Affiliations:** ^1^Pediatric Dentistry, University Nove de Julho (UNINOVE), São Paulo, SP, Brazil; ^2^Rehabilitation Sciences Post Graduation Program, University Nove de Julho (UNINOVE), Rua Vergueiro, 235/249, Liberdade, 01504-001 São Paulo, SP, Brazil; ^3^Bauru School of Dentistry, University of São Paulo, Bauru, SP, Brazil; ^4^Dental Clinic Post Graduation Program, University Federal Fluminense (UFF), Niterói, RJ, Brazil

## Abstract

The aim of the present study was to assess pain and the need for anesthesia during chemomechanical caries removal with Papacarie gel and the traditional method (low-speed bur) in pediatric patients. A randomized, controlled, clinical trial with a “split-mouth” design was carried out involving 20 children (10 girls and 10 boys) aged four to seven years. Forty primary teeth (two per child) were randomly allocated to either Group 1 (G1: chemomechanical caries removal with Papacarie gel) or Group 2 (G2: removal of carious dentin with low-speed bur). A face scale was used to classify the sensation of pain during the procedure (1: absence of pain; 2: mild pain; 3: moderate pain; 4: moderately intense pain; 5: intense pain; and 6: extremely intense pain). Statistical analysis of the data was performed using the Wilcoxon-Mann-Whitney (*U*) test. Pain scores were higher in G2, with statistically significant differences in comparison to G1 (*U* = 148.0; *W* = 358.0; *P* = 0.041). Chemomechanical caries removal with Papacarie provides a lesser degree of pain in comparison to conventional caries removal and does not require the use of local anesthesia. The clinical trial registration number is NCT01811420.

## 1. Introduction

The aim of minimally invasive restorative treatment in dentistry is the selective removal of carious tissue and the preservation of the maximum amount of sound dental tissue [1–5]. Chemomechanical caries removal (CMCR) is in line with this philosophy and consists of the application of a proteolytic substance to soften carious dentin and facilitate the removal of the affected tissue with the use of blunt instruments [3, 6–8].

Papacarie gel is a product designed for CMCR. This gel unites the cleaning and healing (antibacterial and anti-inflammatory) properties of papain with the disinfecting properties of chloramine [9]. Studies have demonstrated the efficacy of this product [6, 7, 10, 11] and emphasize its use on children [6, 12, 13], adolescents [7], and individuals with disabilities [14].

The removal of carious lesions using a low-speed bur (conventional method) involves physical and mechanical stimuli that cause pain [3]. In young patients, anxiety regarding dental treatment due to the possibility of experiencing pain hampers this type of procedure [15]. Moreover, studies have reported that noninvasive methods cause less discomfort to patients [12, 16, 17].

The aim of the present study was to assess pain and the need for anesthesia during chemomechanical caries removal with Papacarie gel and the conventional method (low-speed bur) in pediatric patients.

## 2. Material and Methods

This study received approval from the Human Research Ethics Committee of *Universidade Nove de Julho* (Brazil) under process number 219047. All legal guardians received information on the objectives of the study and agreed to the participation of the children by signing a statement of informed consent in compliance with Resolution 196/96 of the Brazilian National Board of Health. The clinical trial registration number is NCT01811420.

A randomized, controlled, clinical trial with a “split-mouth” design was carried out involving 20 children (10 girls and 10 boys) aged four to seven years. The sample size was calculated using the Dinamalan 1.0 program and based on data from a pilot study. The power of the sample is the probability of finding different results between the groups. Calculating the power of sample, we got 19 teeth per group, considering a significance level of 5% (*P* < 0.05) on the analysis. The procedures were performed by a single operator who had undergone a calibration exercise (Kappa = 0.9). The calibration was carried out in a previous study in which the operator performed the treatment and an examiner (“gold standard”) performed the evaluation of the treatment. The examiner was blinded to the techniques applied and evaluated all cavities after the respective interventions. The examiner was also responsible for testing the hardness of the remaining dentin. Adequate agreement was required between the operator and examiner before the removal of the carious dentin was considered concluded. This agreement was determined using the Kappa statistic.

**Figure 1:**
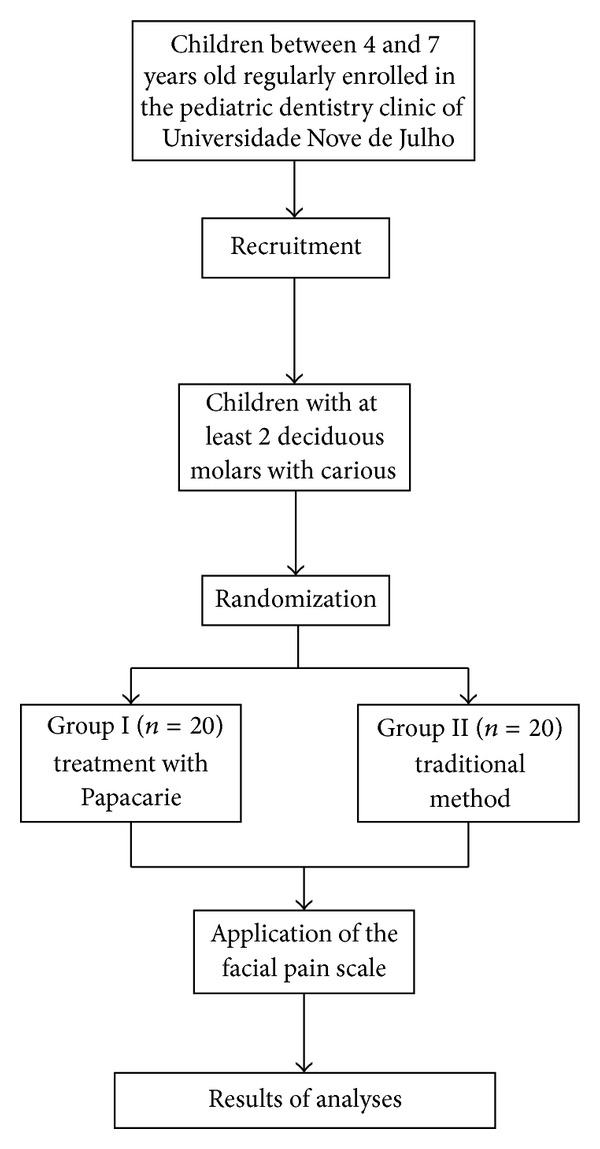
Flow chart of the protocol.

The investigation was designed, analyzed, and interpreted according to the Consolidated Standards of Reporting Trials (CONSORT) (Figure 1). Individuals with no systemic conditions, adequate behavior, and at least two primary molars with active, acute carious lesions on the occlusal face not surpassing 2/3 of the dentin, with direct visualization and access and no clinical or radiographic signs or symptoms of pulp involvement, were included in the study. In case of an individual with more than two molars meeting the inclusion criteria, a raffle was done to select only two. The teeth were randomly allocated by lots to either Group 1 (G1: chemomechanical caries removal (CMCR) with Papacarie gel; number of teeth involved = 20) or Group 2 (G2: removal of carious lesion with using a low-speed bur; number of teeth involved = 20). Randomization was performed by lots using numbered tiles to determine the tooth and the treatment that would be done first. The other tooth in the same subject was automatically submitted to the other form of treatment.

G1 was first submitted to periapical and interproximal duo film radiography and prophylaxis with a Robinson brush and fluoride toothpaste, followed by relative isolation with a lip bumper, cotton roll, and aspirator. Papacarie gel was applied and allowed to stand for 30 to 40 seconds, followed by mechanical removal of the softened carious tissue with the blunt end of an excavator. The gel was reapplied, if necessary, until all carious tissue was removed. The determination of the completion of the process was based on the coloration of the gel (clean and without debris) and hardness of the remaining dentin (sufficient to impede the penetration of the exploratory probe with a rhombus tip). The restorations were performed with glass ionomer cement (Ketac Molar Easy Mix—3 m ESPE). In a different visit, G2 was submitted to the same procedures as G1, with the exception of the caries removal procedure, which was performed using a low-speed bur.

Both procedures were initiated without the prior administration of local anesthesia. After the caries removal, the patients were instructed to select an image from a face scale to classify the sensation of pain during the procedure (0: absence of pain; 1: mild pain; 2: moderate pain; 3: moderately intense pain; 4: intense pain; and 5: extremely intense pain) [18, 19]. In the presence of pain during the procedure, the face scale would be used and when the score was ≥3, the need for the administration of anesthesia was based on the evaluation of the procedure, patient behavior, and the cause of the pain.

The nonparametric Wilcoxon-Mann-Whitney (*U*) test was used to test the hypothesis that G1 would have a lower degree of pain (as determined by the pain scale) due to the lesser physical and mechanical stimuli in comparison to G2. The statistical analysis was performed using the SPSS program (v. 12.0, SPSS Inc., Chicago, IL, USA) with the level of significance set to 5% (*P* < 0.05).

## 3. Results

The sample was made up of 20 children (12 girls and 8 boys) aged four to seven years (mean age: 5.6 years) (Table 1).  Table 2 displays the results of the administration of the pain scale. One case received a score denoting moderate pain (2) and one case received a score denoting moderately intense pain (3) and required the administration of local anesthesia, both during treatment with bur (G2). The scores were also evaluated by gender, and no statistically significant difference was found (Table 3). Moreover, a significantly greater amount of pain was found in the group submitted to conventional treatment (*U* = 148.0; *W* = 358.0; *P* = 0.041).

## 4. Discussion

The findings demonstrated that children submitted to conventional caries removal with a low-speed bur experienced greater pain in comparison to those submitted to CMCR, with statistical significance. The greater degree of pain in the former group may be attributed to effect that burs tend to exert on dental structures [3]. The present findings are in agreement with data reported in previous studies [12, 13, 17]. In one of the investigations cited, the authors point out the viability of the use of Papacarie within the precepts of minimally invasive dentistry, as this technique allows greater patient comfort and an absence of pain [13].

The degree of pain during the removal of the carious lesions was determined using a face scale, which revealed the absence of pain (<3) in the group submitted to CMCR. In contrast, the group submitted to conventional treatment reported higher degrees of pain. The use of the face scale was based on its ease of understanding among individuals in the age group studied and on the fact that this scale has been widely employed in studies assessing pain [20, 21]. This method for assessing pain demonstrates the importance of alternatives that consider the perceptions of children, who often have not had prior experience with dental pain and require an assessment method that is easy to comprehend [22].

Metal burs coupled to low-speed hand-held rotary instruments have been the most widely employed tools for the removal of carious lesions. However, this method can cause pain and may require the need for local anesthesia [23]. Studies have investigated this issue by comparing the conventional method with techniques in line with the philosophy of minimally invasive treatment. In one such study, the authors determined the use of local anesthesia based on (1) the degree of patient pain, (2) the decision on the part of the dentist which aimed at providing greater patient comfort and safety, or (3) an effort to avoid negative behavior during the procedure. The results demonstrated no statistically significant differences between methods regarding the need for anesthesia [24]. In the present study, only one case required anesthesia, which was based on the score the patient attributed to the intensity of pain experienced. This occurred during the conventional treatment procedure (low-speed bur), which is in agreement with findings described in a study by De Menezes Abreu et al. (2011) [15]. Pain tends to be lesser with the use of Papacarie due to the preservation of sound dental tissue during the procedure, which is not possible with the conventional method [6, 12]. Indeed, caries removal with a low-speed bur also involves the removal of sound dental tissue and increases the risk of pulp injury [25].

## 5. Conclusion

Based on the present findings, chemomechanical caries removal with Papacarie provides a lesser degree of pain in comparison to conventional caries removal and does not require the use of local anesthesia.

## Figures and Tables

**Table 1:** Distribution of children in sample according to age.

Age	*n*	%
4	5	25.0%
5	5	25.0%
6	3	15.0%
7	7	35.0%

Total	20	100.0%

**Table 2:** Frequency of scores given by children for pain intensity using face scale.

Score	Bur *n* (%)	CMCR *n* (%)	*P* value
0 (no pain)	13 (65.0)	18 (90.0)	*P* = 0.041
1 (mild)	5 (25.0)	2 (10.0)	
2 (moderate)	1 (5.0)	0 (0.0)	
3 (moderately intense)	1 (5.0)	0 (0.0)	
4 (intense)	0 (0.0)	0 (0.0)	
5 (extremely intense)	0 (0.0)	0 (0.0)	

Total	20 (100.0)	20 (100.0)	

**Table 3:** Frequency of scores given by children.

	Score	Girls (%)	Boys (%)	*P* value
Group 1	0 (no pain)	12 (100.0)	6 (75.0)	*P* = 0.075^a^
	1 (mild)	0 (0.0)	2 (25.0)	
	2 (moderate)	0 (0.0)	0 (0.0)	
	3 (moderately intense)	0 (0.0)	0 (0.0)	
	4 (intense)	0 (0.0)	0 (0.0)	
	5 (extremely intense)	0 (0.0)	0 (0.0)	

	Total	12 (100.0)	8 (100.0)	

Group 2	0 (no pain)	10 (83.3)	3 (37.5)	*P* = 0.055^b^
	1 (mild)	1 (8.3)	4 (50.0)	
	2 (moderate)	1 (8.3)	0 (0.0)	
	3 (moderately intense)	0 (0.0)	1 (12.5)	
	4 (intense)	0 (0.0)	0 (0.0)	
	5 (extremely intense)	0 (0.0)	0 (0.0)	

	Total	12 (100.0)	8 (100.0)	

^a^Mann-Whitney, *U* = 36,000. ^b^Mann-Whitney, *U* = 27,000
